# Editorial: Chemical sensing and emerging analysis of environmental contaminants

**DOI:** 10.3389/fchem.2023.1348108

**Published:** 2023-12-11

**Authors:** Shengshui Hu, Cheng Fang

**Affiliations:** ^1^ College of Chemistry and Molecule Science, Wuhan University, Wuhan, China; ^2^ Global Centre for Environmental Remediation (GCER), University of Newcastle, Callaghan, NSW, Australia; ^3^ CRC for Contamination Assessment and Remediation of the Environment (CRC CARE), University of Newcastle, Callaghan, NSW, Australia

**Keywords:** PFAS, microplastic, mercury, chemical sensing, environmental contaminant analysis

## Introduction

In the dynamic landscape of environmental science, our collective pursuit of understanding and mitigating environmental contaminants takes centre stage in this Research Topic on “*Chemical Sensing and Emerging Analysis of Environmental Contaminants*.” This Research Topic of articles transcends traditional boundaries, offering a comprehensive exploration of innovative methodologies, advanced technologies and molecular-level insights. In this Editorial, we navigate through the contributions of diverse studies, each playing a unique role in expanding our comprehension of environmental challenges.

## Framing the aims and objectives

The overarching aim of this Research Topic is to advance the field of chemical sensing and analysis, focusing on some merged and emerging environmental contaminants. As we delve into the contributing articles, our objective is clear: to harness cutting-edge techniques and methodologies that empower us to detect and understand environmental contaminants with unprecedented precision. From the molecular detection to the application of novel nanotechnologies, each article contributes to a collective effort to enhance our analytical capabilities in the realm of environmental science.

## Contributing articles: a mosaic of analytical insights

Our exploration commences with a refined focus on an emerging contaminant of per- and polyfluoroalkyl substances (PFAS), where a modified Total Oxidable Precursor (TOP) assay proves to be effective to unveil the unknown PFAS precursors Amin et al. This article sets the stage by enhancing the detection methodologies, illustrating the importance of precision in understanding PFAS’ presence and impact in the environment. Our journey also culminates in a molecular detection approach, utilising time-of-flight secondary ion mass spectrometry (ToF-SIMS) to identify PFASs in water Yu et al. This sophisticated technique provides molecular-level insights, offering a streamlined solution for environmental forensic analysis of PFASs in wastewater. After many years of research on PFAS, the detection is still a challenge and more research is needed.

Moving forward, the journey takes us into the microscopic world of microplastics and nanoplastics released from kitchen sealants, where advanced Raman imaging becomes a powerful tool for environmental monitoring Fang et al. This work not only identifies contaminants but also highlights the role of sophisticated imaging techniques in uncovering hidden threats. The narrative seamlessly transitions to the realm of flow cytometry, offering the refined detection protocols for microplastics and nanoplastics Li et al. This article, too, underscores the significance of innovative analytical techniques, providing a clearer picture of emerging contaminants in environmental matrices. So far, there is absent of the standardised monitoring approach for microplastics, even less for nanoplastics, we actually have a very limited knowledge about their sources, fate, toxicity and risk.

The synthesis of vancomycin-functionalized fluorescent gold nanoparticles takes centre stage as well, introducing a highly selective assay for the detection of mercury (II) Tiwari et al. This work not only addresses the urgency of mercury detection, simplifies the detection protocol, but also showcases the potential of nanotechnology in precise environmental monitoring.

## Placing findings in a broader context

As we reflect on these contributions, the broader context comes into focus. The amalgamation of analytical techniques, chemometrics (such as principal component analysis PCA to analyse hyper spectrum of Raman or mass spectrum), nanotechnologies, and molecular-level investigations highlights the multidisciplinary nature of chemical sensing in environmental science. Each article, while contributing unique insights, collectively reinforces the interconnectedness of environmental challenges and the need for a holistic approach to analytical methodologies.

## In conclusion: paving the way forward

This Research Topic not only signifies individual achievements but also charts a course for the future of chemical sensing in environmental science. The refined detection methodologies, innovative imaging techniques, and molecular-level insights presented in these articles collectively pave the way for a more comprehensive understanding of environmental contaminants. As we absorb the knowledge encapsulated in these studies, we are reminded that the quest for environmental awareness is an evolving narrative, with each study adding a layer to the story of chemical sensing and environmental analysis.

